# Supplementary material for: Lipidomic Profiling of Rice Bran after Green Solid–Liquid Extractions for the Development of Circular Economy Approaches
Source: Foods. 2023 Jan 13;12(2):384. doi: 10.3390/foods12020384 (PMC9857567; doi:10.3390/foods12020384)
Supplement: Supplementary file 1 [file foods-12-00384-s001.zip › Table S1.pdf]

Table S1

Legend	
**Abbreviation**	**Name**
**ET**	Ethanol (99%) at 4 °C
**ET20**	Ethanol (99%) at 20 °C
**WSBU**	Water-saturated 1-butanol at 4 °C
**CH-ME**	Chloroform/methanol (2:1, v/v) at 4 °C
**MTBE-ME**	Methyl tert-butyl ether/methanol (3:1, v/v) at 4 °C
**LPC**	Lysophosphatidylcholine
**LPE**	Lysophosphatidylethanolamine
**PC**	Phosphatidylcholine
**PE**	Phosphatidylethanolamine
**PG**	Phosphoglycerol
**PI**	Phosphatidylinositols
**LPLs**	Lysophospholipids
**PLs**	Phospholipids

The Relative Abundances of PLs and LPLs in different methods

Name	ET SAMPLE 1	ET SAMPLE 2	ET20 SAMPLE 1	ET20 SAMPLE 2	WSBU SAMPLE 1	WSBU SAMPLE 2	CH-ME SAMPLE 1	CH-ME SAMPLE 2	MTBE-ME SAMPLE 1	MTBE-ME SAMPLE 2
**LPC 16:0**	207448.27	204945.52	0.00	0.00	290257.28	317502.27	0.00	0.00	0.00	0.00
**LPC 16:0/0:0**	610171.41	564635.93	662080.88	624667.73	1648420.23	1540908.29	456587.36	705844.53	1024748.33	1503866.70
**LPC 18:1**	159436.22	150015.57	8302255.53	7520207.74	9508539.19	9115664.12	3744393.59	7016758.59	222199.83	325926.84
**LPC 18:1/0:0**	4159406.27	3484195.05	3284619.01	3230213.70	5045655.95	7381328.62	2433985.66	2675835.94	5102592.89	7066326.99
**LPC 18:2**	233053.68	231220.21	240682.76	218543.98	6593081.97	6932425.80	2941491.13	4485630.63	4701720.89	7024048.74
**LPC 18:2/0:0**	2609000.22	2395782.89	2489403.09	2199537.68	3765810.87	5173208.84	1381480.85	2094960.94	3138423.78	4335105.24
**LPC 18:3**	155817.62	149544.90	0.00	0.00	170373.87	180895.64	0.00	0.00	97761.06	149245.58
**LPE 16:0**	0.00	0.00	0.00	0.00	0.00	0.00	0.00	0.00	107185.83	160302.18
**LPE 18:1**	400596.22	360900.10	348765.75	358015.58	324396.24	353623.51	0.00	0.00	459937.33	757298.74
**LPE 18:2**	725748.49	696271.03	662499.45	666748.23	563299.36	624601.30	229156.60	325011.09	849264.39	1203696.02
**LPE O-3:0**	18536375.35	18015665.15	18857104.97	17950118.90	4411133.87	4204102.10	0.00	0.00	0.00	0.00
**PC 32:0**	319468.49	313456.80	111711.27	309228.95	0.00	0.00	230309.62	347010.78	613093.06	1002635.34
**PC 32:0|PC 16:0_16:0**	0.00	0.00	0.00	0.00	346042.31	222142.60	0.00	0.00	0.00	0.00
**PC 32:1**	473897.62	222372.99	397638.12	462347.79	482903.99	530347.51	503508.40	439250.78	804585.94	1369851.94
**PC 32:2**	159856.59	291861.13	348358.73	289137.57	323913.87	337099.45	257968.49	436223.75	762006.89	906660.49
**PC 34:1**	505832.86	505371.13	12334275.30	11661935.64	284878.61	592106.30	389053.68	620453.13	2047088.00	1141140.39
**PC 34:1|PC 16:0_18:1**	11404672.86	11188638.35	0.00	0.00	7695310.98	12593889.94	9608360.00	14501408.75	20035011.56	35597284.66
**PC 34:2**	414905.08	385886.60	10374440.06	9033438.79	423031.79	464287.62	7996020.76	12133611.56	1527399.00	682354.47
**PC 34:2|PC 16:0_18:2**	9182547.89	8635185.16	0.00	0.00	8939665.20	9708064.53	0.00	0.00	24825409.78	26492551.46
**PC 34:3**	254302.65	228260.93	285322.71	243616.24	232586.59	236378.18	222682.55	352814.38	770296.67	330822.04
**PC 36:1**	488558.97	460385.52	522938.45	475525.47	514104.39	551470.72	373692.08	465530.70	1745051.44	1572850.10
**PC 36:2**	524793.68	532705.77	538503.15	519631.05	612292.49	582283.04	388855.09	573907.34	2221025.56	2426666.60
**PC 36:2|PC 18:1_18:1**	10880218.81	10336839.59	10812670.94	10421810.39	10799417.34	12418347.85	7736520.76	11998945.00	34125223.11	36624105.63
**PC 36:3**	15724753.89	16008057.58	900458.12	760205.30	853395.14	880288.56	562969.43	1049637.03	3532820.22	4058672.23
**PC 36:3|PC 18:1_18:2**	0.00	0.00	16355546.52	10325893.48	16878692.25	15978430.94	12966710.94	18955680.00	47026453.33	48982219.81
**PC 36:4**	342572.86	315874.07	397457.90	183898.62	312547.34	324222.43	286469.43	462599.38	1029642.78	1117684.27
**PC 36:4|PC 18:2_18:2**	5208272.43	6831839.18	8332803.54	6687465.19	6709515.38	7284950.28	6150366.42	10026608.13	18745905.78	20012542.14
**PC 36:5**	178490.59	380981.29	427300.88	413889.61	184289.60	278774.09	363584.43	637058.59	918790.06	987623.50
**PC 38:1**	0.00	0.00	116222.93	96486.52	0.00	0.00	0.00	0.00	478961.72	559761.80
**PC 37:2**	0.00	0.00	0.00	0.00	0.00	0.00	520593.77	455217.58	0.00	0.00
**PC 38:2**	0.00	0.00	0.00	0.00	0.00	0.00	0.00	0.00	387295.67	411858.40
**PC 38:3**	0.00	0.00	0.00	0.00	0.00	0.00	0.00	0.00	353114.61	387486.17

Name	ET SAMPLE 1	ET SAMPLE 2	ET20 SAMPLE 1	ET20 SAMPLE 2	WSBU SAMPLE 1	WSBU SAMPLE 2	CH-ME SAMPLE 1	CH-ME SAMPLE 2	MTBE-ME SAMPLE 1	MTBE-ME SAMPLE 2
PC 42:1	0.00	0.00	0.00	0.00	109783.99	130818.12	0.00	0.00	368727.33	328417.09
PC 42:2	0.00	0.00	0.00	0.00	0.00	0.00	0.00	0.00	404800.33	377251.84
PC 44:1	0.00	0.00	0.00	0.00	0.00	0.00	0.00	0.00	113446.72	106682.82
PC O-44:1	0.00	0.00	641068.34	441426.52	0.00	0.00	0.00	0.00	498972.44	459204.51
PE 32:2	0.00	0.00	0.00	0.00	0.00	0.00	0.00	0.00	180222.11	168199.32
PE 34:1	0.00	0.00	0.00	0.00	0.00	0.00	0.00	0.00	563873.06	500262.67
PE 34:1|PE 16:0_18:1	961678.49	989963.40	873905.69	910576.13	1116837.69	1128531.27	504187.36	732992.73	4407671.11	4551329.32
PE 34:2	221822.16	216743.71	208390.33	218357.35	242832.72	222911.99	0.00	0.00	820845.28	787360.92
PE 34:2|PE 16:0_18:2	2394530.38	2213462.47	2160895.03	2335810.83	2319018.27	2730138.34	839362.36	1307932.50	10182342.22	10989607.77
PE 36:2	0.00	0.00	0.00	0.00	0.00	0.00	0.00	0.00	339048.94	345257.62
PE 36:2|PE 18:1_18:1	526313.51	546219.38	566403.09	546065.30	599184.05	564350.11	273715.47	436695.31	2796517.33	2557509.71
PE 36:3	0.00	0.00	0.00	0.00	0.00	0.00	0.00	0.00	625674.61	636249.66
PE 36:3|PE 18:1_18:2	1430587.46	1254389.07	1362612.04	808644.92	1381317.46	1493152.16	805151.70	1288752.03	6111664.89	6291059.81
PE 36:4	0.00	0.00	0.00	0.00	0.00	0.00	0.00	0.00	451928.44	430131.70
PE 36:4|PE 18:2_18:2	1007730.49	993015.36	1054101.66	1003462.98	508733.12	872387.79	819653.87	1211477.58	3478487.78	3757489.32
PE 42:2	0.00	0.00	0.00	0.00	0.00	0.00	0.00	0.00	189924.39	170371.17
PG 34:2	0.00	0.00	0.00	0.00	0.00	0.00	0.00	0.00	131138.67	162486.89
PI 34:1	201579.95	237109.07	198681.22	211341.44	408821.39	348078.23	0.00	0.00	475648.06	508688.88
PI 34:2	496292.27	509000.88	476138.29	485215.30	944343.82	836299.72	0.00	0.00	1002189.11	1010641.46
PI 36:2	0.00	0.00	0.00	0.00	0.00	0.00	0.00	0.00	140364.33	152909.42
PI 36:3	84948.43	148547.47	149025.86	66215.69	276005.78	256213.70	0.00	0.00	303461.44	354595.83
PI 36:4	0.00	0.00	0.00	0.00	150812.08	151449.94	0.00	0.00	165614.06	185022.72
