# Supplementary material for: Lipidomic Profiling of Rice Bran after Green Solid–Liquid Extractions for the Development of Circular Economy Approaches
Source: Foods. 2023 Jan 13;12(2):384. doi: 10.3390/foods12020384 (PMC9857567; doi:10.3390/foods12020384)
Supplement: Supplementary file 1 [file foods-12-00384-s001.zip › Table S2.pdf]

Table S2

Legend	
**Abbreviation**	**Name**
**ET**	Ethanol (99%) at 4 °C
**ET20**	Ethanol (99%) at 20 °C
**WSBU**	Water-saturated 1-butanol at 4 °C
**CH-ME**	Chloroform/methanol (2:1, v/v) at 4 °C
**MTBE-ME**	Methyl tert-butyl ether/methanol (3:1, v/v) at 4 °C
**Cer**	Ceramide
**HexCer**	Hexosylceramide

The Relative Abundances of Cers and HexCers in different methods

<table>
<tr><th>Name</th><th>ET SAMPLE 1</th><th>ET SAMPLE 2</th><th>ET20 SAMPLE 1</th><th>ET20 SAMPLE 2</th><th>WSBU SAMPLE 1</th><th>WSBU SAMPLE 2</th><th>CH-ME SAMPLE 1</th><th>CH-ME SAMPLE 2</th><th>MTBE-ME SAMPLE 1</th><th>MTBE-ME SAMPLE 2</th></tr>
<tr><td>Cer 40:0;4O|Cer 18:0;3O/22:0;(2OH)</td><td>0.00</td><td>0.00</td><td>0.00</td><td>0.00</td><td>135587.57</td><td>176370.66</td><td>0.00</td><td>0.00</td><td>380502.22</td><td>392615.00</td></tr>
<tr><td>Cer 40:1;4O|Cer 18:1;3O/22:0;(2OH)</td><td>0.00</td><td>0.00</td><td>0.00</td><td>0.00</td><td>0.00</td><td>0.00</td><td>0.00</td><td>0.00</td><td>208519.78</td><td>226813.50</td></tr>
<tr><td>Cer 41:0;4O|Cer 18:0;3O/23:0;(2OH)</td><td>0.00</td><td>0.00</td><td>0.00</td><td>0.00</td><td>0.00</td><td>0.00</td><td>0.00</td><td>0.00</td><td>375094.89</td><td>331253.98</td></tr>
<tr><td>Cer 41:1;4O|Cer 18:1;3O/23:0;(2OH)</td><td>0.00</td><td>0.00</td><td>0.00</td><td>0.00</td><td>0.00</td><td>0.00</td><td>0.00</td><td>0.00</td><td>178585.06</td><td>203617.48</td></tr>
<tr><td>Cer 42:0;4O|Cer 18:0;3O/24:0;(2OH)</td><td>575356.32</td><td>554892.63</td><td>599982.82</td><td>660923.43</td><td>809050.75</td><td>1220857.46</td><td>645441.42</td><td>550680.00</td><td>2648474.22</td><td>2666060.97</td></tr>
<tr><td>Cer 42:1;4O|Cer 18:1;3O/24:0;(2OH)</td><td>0.00</td><td>0.00</td><td>0.00</td><td>0.00</td><td>279010.17</td><td>394061.33</td><td>0.00</td><td>0.00</td><td>899282.94</td><td>829390.49</td></tr>
<tr><td>Cer 43:0;4O|Cer 18:0;3O/25:0;(2OH)</td><td>229182.70</td><td>196583.40</td><td>249273.48</td><td>229201.55</td><td>286765.90</td><td>427198.45</td><td>0.00</td><td>0.00</td><td>929968.83</td><td>853800.97</td></tr>
<tr><td>Cer 44:0;2O|Cer 20:0;2O/24:0</td><td>354303.30</td><td>190182.06</td><td>0.00</td><td>0.00</td><td>384517.98</td><td>353538.12</td><td>0.00</td><td>0.00</td><td>0.00</td><td>0.00</td></tr>
<tr><td>Cer 44:0;4O|Cer 18:0;3O/26:0;(2OH)</td><td>426883.62</td><td>407077.53</td><td>390037.68</td><td>401188.84</td><td>529438.38</td><td>626748.40</td><td>375674.06</td><td>334805.94</td><td>1194302.89</td><td>1106950.10</td></tr>
<tr><td>Cer 44:1;4O|Cer 18:1;3O/26:0;(2OH)</td><td>0.00</td><td>0.00</td><td>0.00</td><td>0.00</td><td>0.00</td><td>0.00</td><td>0.00</td><td>0.00</td><td>225303.33</td><td>213770.44</td></tr>
<tr><td>Cer 46:0;2O|Cer 20:0;2O/26:0</td><td>0.00</td><td>0.00</td><td>0.00</td><td>0.00</td><td>345884.05</td><td>361464.59</td><td>0.00</td><td>0.00</td><td>0.00</td><td>0.00</td></tr>
<tr><td>Cer 46:0;2O|Cer 22:0;2O/24:0</td><td>436454.27</td><td>353059.59</td><td>343270.55</td><td>322152.38</td><td>0.00</td><td>0.00</td><td>870186.23</td><td>563987.73</td><td>365964.06</td><td>289323.20</td></tr>
<tr><td>Cer 47:1;4O|Cer 22:0;3O/25:1;(2OH)</td><td>157170.92</td><td>170879.18</td><td>0.00</td><td>0.00</td><td>0.00</td><td>0.00</td><td>0.00</td><td>0.00</td><td>0.00</td><td>0.00</td></tr>
<tr><td>Cer 48:0;2O|Cer 24:0;2O/24:0</td><td>0.00</td><td>0.00</td><td>0.00</td><td>0.00</td><td>0.00</td><td>0.00</td><td>0.00</td><td>0.00</td><td>0.00</td><td>0.00</td></tr>
<tr><td>Cer 48:0;2O|Cer 22:0;2O/26:0</td><td>0.00</td><td>0.00</td><td>0.00</td><td>0.00</td><td>0.00</td><td>0.00</td><td>772712.08</td><td>356242.50</td><td>0.00</td><td>0.00</td></tr>
<tr><td>HexCer 38:2;3O|HexCer 18:2;2O/20:0;O</td><td>0.00</td><td>0.00</td><td>0.00</td><td>0.00</td><td>0.00</td><td>0.00</td><td>0.00</td><td>0.00</td><td>420342.33</td><td>466767.18</td></tr>
<tr><td>HexCer 40:1;4O|HexCer 18:1;3O/22:0;(2OH)</td><td>0.00</td><td>0.00</td><td>0.00</td><td>0.00</td><td>0.00</td><td>0.00</td><td>0.00</td><td>0.00</td><td>467706.83</td><td>405640.97</td></tr>
<tr><td>HexCer 42:1;4O|HexCer 18:1;3O/24:0;(2OH)</td><td>0.00</td><td>0.00</td><td>0.00</td><td>0.00</td><td>170182.89</td><td>160150.39</td><td>0.00</td><td>0.00</td><td>786978.17</td><td>735813.16</td></tr>
<tr><td>HexCer 42:2;3O|HexCer 18:2;2O/24:0;O</td><td>0.00</td><td>0.00</td><td>0.00</td><td>0.00</td><td>0.00</td><td>0.00</td><td>0.00</td><td>0.00</td><td>162154.00</td><td>150371.50</td></tr>
<tr><td>HexCer 44:1;4O|HexCer 18:1;3O/26:0;(2OH)</td><td>0.00</td><td>0.00</td><td>0.00</td><td>0.00</td><td>0.00</td><td>0.00</td><td>0.00</td><td>0.00</td><td>146585.56</td><td>140688.35</td></tr>
</table>
