# Supplementary material for: Lipidomic Profiling of Rice Bran after Green Solid–Liquid Extractions for the Development of Circular Economy Approaches
Source: Foods. 2023 Jan 13;12(2):384. doi: 10.3390/foods12020384 (PMC9857567; doi:10.3390/foods12020384)
Supplement: Supplementary file 1 [file foods-12-00384-s001.zip › Table S3.pdf]

# Table S3

| Legend | |
|---|---|
| **Abbreviation** | **Name** |
| **ET** | Ethanol (99%) at 4 °C |
| **ET20** | Ethanol (99%) at 20 °C |
| **WSBU** | Water-saturated 1-butanol at 4 °C |
| **CH-ME** | Chloroform/methanol (2:1, v/v) at 4 °C |
| **MTBE-ME** | Methyl tert-butyl ether/methanol (3:1, v/v) at 4 °C |
| **DG** | Diglyceride |
| **TG** | Triglyceride |

## The Relative Abundances of DGs and TGs in different methods

| Name | ET SAMPLE 1 | ET SAMPLE 2 | ET20 SAMPLE 1 | ET20 SAMPLE 2 | WSBU SAMPLE 1 | WSBU SAMPLE 2 | CH-ME SAMPLE 1 | CH-ME SAMPLE 2 | MTBE-ME SAMPLE 1 | MTBE-ME SAMPLE 2 |
|---|---|---|---|---|---|---|---|---|---|---|
| **DG 26:1** | 0.00 | 0.00 | 0.00 | 0.00 | 0.00 | 0.00 | 0.00 | 0.00 | 0.00 | 0.00 |
| **DG 28:2** | 122837.89 | 173181.13 | 0.00 | 0.00 | 103112.77 | 72809.12 | 0.00 | 0.00 | 360843.83 | 57168.59 |
| **DG 30:5** | 0.00 | 0.00 | 0.00 | 0.00 | 0.00 | 0.00 | 0.00 | 0.00 | 234441.89 | 130439.90 |
| **DG 31:3** | 0.00 | 0.00 | 0.00 | 0.00 | 29686.76 | 153059.23 | 0.00 | 0.00 | 0.00 | 0.00 |
| **DG 32:0** | 1516932.87 | 1318145.05 | 1196795.91 | 1360996.80 | 569098.32 | 841691.38 | 0.00 | 0.00 | 1896995.11 | 1615953.88 |
| **DG 32:0\|DG 16:0_16:0** | 0.00 | 0.00 | 0.00 | 0.00 | 0.00 | 0.00 | 0.00 | 0.00 | 220086.94 | 198383.59 |
| **DG 32:1** | 887188.43 | 707125.05 | 649745.41 | 616417.79 | 625241.97 | 1002099.01 | 0.00 | 0.00 | 1150742.89 | 1100915.63 |
| **DG 32:2** | 1473552.54 | 1276276.91 | 522126.69 | 1223704.53 | 1087936.65 | 1604642.65 | 0.00 | 0.00 | 2537418.44 | 2617317.09 |
| **DG 32:2\|DG 14:0_18:2** | 228637.95 | 370382.89 | 172732.87 | 341944.20 | 157976.99 | 463309.34 | 0.00 | 0.00 | 680575.00 | 696242.52 |
| **DG 33:2** | 267004.00 | 171252.84 | 0.00 | 0.00 | 168282.72 | 256037.90 | 0.00 | 0.00 | 292748.94 | 355937.77 |
| **DG 33:3** | 120860.22 | 172441.86 | 0.00 | 0.00 | 150671.73 | 248849.89 | 0.00 | 0.00 | 326271.50 | 311569.32 |
| **DG 33:3\|DG 15:1_18:2** | 0.00 | 0.00 | 0.00 | 0.00 | 0.00 | 0.00 | 0.00 | 0.00 | 236045.11 | 265762.52 |
| **DG 34:0** | 278477.24 | 235618.04 | 205422.87 | 203494.81 | 3755871.94 | 5525305.97 | 0.00 | 0.00 | 274526.33 | 208266.60 |
| **DG 34:1** | 36478834.16 | 31389268.45 | 31254064.09 | 32419931.93 | 12366031.75 | 17481616.10 | 0.00 | 0.00 | 43937077.33 | 42337168.16 |
| **DG 34:1\|DG 16:0_18:1** | 19459367.78 | 16521276.70 | 14761336.57 | 16475672.04 | 12983322.08 | 20339716.24 | 0.00 | 0.00 | 22820048.00 | 23062073.79 |
| **DG 34:2** | 70657286.92 | 63961646.19 | 59472251.05 | 64794469.83 | 20037449.69 | 27323895.65 | 0.00 | 0.00 | 94488049.78 | 86321590.68 |
| **DG 34:2\|DG 16:0_18:2** | 68525747.89 | 57893733.61 | 52419200.00 | 60776952.93 | 51873975.49 | 75384298.78 | 0.00 | 0.00 | 90284053.33 | 86645735.15 |
| **DG 34:2\|DG 16:1_18:1** | 222764.49 | 166017.32 | 0.00 | 0.00 | 125176.07 | 224910.72 | 0.00 | 0.00 | 272324.33 | 224815.92 |
| **DG 34:3** | 3078016.87 | 2854536.91 | 2304560.66 | 2608158.90 | 1669530.78 | 2348661.60 | 251108.11 | 439695.31 | 4628077.78 | 4308884.27 |
| **DG 34:3\|DG 16:0_18:3** | 2796292.32 | 2431881.65 | 1946701.66 | 2233399.78 | 2036668.90 | 2987292.82 | 0.00 | 0.00 | 4335088.44 | 4230321.55 |
| **DG 34:3\|DG 16:1_18:2** | 730784.59 | 559071.39 | 427039.34 | 555455.47 | 455292.54 | 764568.34 | 0.00 | 0.00 | 996012.11 | 969897.86 |
| **DG 35:3** | 0.00 | 0.00 | 0.00 | 0.00 | 0.00 | 0.00 | 0.00 | 0.00 | 122106.22 | 202141.50 |
| **DG 35:4\|DG 17:2_18:2** | 0.00 | 0.00 | 0.00 | 0.00 | 0.00 | 0.00 | 0.00 | 0.00 | 167116.44 | 181545.29 |
| **DG 36:1** | 3359564.76 | 2863583.92 | 2609345.41 | 2627581.22 | 2171421.73 | 2837608.40 | 246999.62 | 369778.20 | 3335989.11 | 2635561.75 |
| **DG 36:1\|DG 18:0_18:1** | 1446565.30 | 1203896.29 | 1023272.60 | 1113722.98 | 920985.72 | 1230456.13 | 0.00 | 0.00 | 1467771.56 | 1193065.83 |
| **DG 36:2** | 7198059.24 | 7796072.58 | 69603168.18 | 66945294.14 | 36152536.18 | 45314232.93 | 7849624.15 | 13013170.00 | 108907960.90 | 98345301.75 |
| **DG 36:2\|DG 18:0_18:2** | 5761849.08 | 5586331.55 | 4048889.72 | 4170100.33 | 4381503.35 | 5859980.11 | 0.00 | 0.00 | 0.00 | 0.00 |
| **DG 36:2\|DG 18:1_18:1** | 94409222.92 | 82037951.34 | 79159103.65 | 79361329.50 | 70606457.34 | 97479361.77 | 5175268.68 | 7537375.00 | 123655153.80 | 118398496.30 |
| **DG 36:3** | 167914980.30 | 149003533.20 | 134577135.00 | 138321057.20 | 130273723.90 | 173971291.90 | 19515761.51 | 32177335.00 | 193232085.30 | 185817003.50 |
| **DG 36:3\|DG 18:1_18:2** | 302303868.50 | 260635500.20 | 222890643.10 | 251289204.00 | 218848037.00 | 317952311.20 | 18907846.04 | 30924660.00 | 372998428.40 | 354978174.80 |
| **DG 36:4** | 13185659.68 | 12614094.02 | 14344305.86 | 11678768.62 | 46011839.08 | 63981043.09 | 6188639.86 | 9803219.61 | 20746828.44 | 19150805.44 |
| **DG 36:4\|DG 18:1_18:3** | 13166784.86 | 12083772.37 | 12923367.96 | 11598123.31 | 9508909.13 | 17813199.56 | 244177.08 | 1463951.72 | 19772016.00 | 18906693.59 |
| **DG 36:4\|DG 18:2_18:2** | 190651336.60 | 168028028.00 | 130483935.50 | 151302053.50 | 136428866.60 | 209479156.70 | 15577596.98 | 21837167.50 | 252092416.00 | 266726797.70 |
| **DG 36:5** | 8059743.14 | 6771256.91 | 6011441.33 | 6652367.74 | 3067298.12 | 4783563.26 | 843153.58 | 919959.38 | 15633633.78 | 15847029.13 |
| **DG 36:5\|DG 18:2_18:3** | 10685866.38 | 9019384.74 | 7662839.34 | 8575467.85 | 8004261.73 | 12562305.41 | 1334267.74 | 2143503.28 | 19781344.00 | 21256542.14 |
| **DG 36:6** | 0.00 | 0.00 | 0.00 | 0.00 | 110820.40 | 175350.39 | 0.00 | 0.00 | 309194.67 | 340744.03 |
| **DG 36:6\|DG 18:3_18:3** | 243110.59 | 206580.00 | 165900.28 | 190240.55 | 200706.94 | 276073.15 | 0.00 | 0.00 | 445370.56 | 512824.13 |
| **DG 37:7** | 1754660.32 | 1841235.05 | 1842608.40 | 3414999.12 | 3661852.95 | 2254005.53 | 3628443.02 | 3330343.13 | 2038173.78 | 1446759.03 |
| **DG 38:1** | 1068718.49 | 968779.18 | 1008117.24 | 916370.55 | 797312.66 | 1119850.94 | 0.00 | 0.00 | 1223746.00 | 940653.01 |
| **DG 38:1\|DG 20:0_18:1** | 819199.08 | 704079.48 | 717522.60 | 654298.78 | 601485.26 | 838223.04 | 0.00 | 0.00 | 900581.44 | 663353.69 |
| **DG 38:10** | 274700.65 | 246903.97 | 260445.86 | 293092.43 | 237103.47 | 320421.24 | 0.00 | 0.00 | 634966.06 | 641852.38 |
| **DG 38:2** | 2642042.60 | 2288264.95 | 2117144.97 | 2304167.29 | 1474456.07 | 1993636.08 | 189769.91 | 288862.97 | 2833248.00 | 2408093.20 |
| **DG 38:2\|DG 18:1_20:1** | 1412246.05 | 1109013.61 | 996819.01 | 1046840.33 | 870353.64 | 1194595.36 | 0.00 | 0.00 | 1341215.67 | 1107800.29 |
| **DG 38:2\|DG 20:0_18:2** | 2500286.27 | 2101765.57 | 1905986.52 | 2062389.17 | 1718348.21 | 2358555.80 | 146121.51 | 254621.09 | 2590589.33 | 2331190.10 |
| **DG 38:3** | 3340144.00 | 3169287.01 | 2645389.17 | 1758956.24 | 2586934.10 | 3515864.75 | 232323.77 | 403665.16 | 4588280.44 | 4028055.15 |
| **DG 38:3\|DG 20:1_18:2** | 3692155.68 | 3418335.26 | 2779306.52 | 1945200.44 | 2741091.79 | 3760956.91 | 241774.15 | 419796.09 | 5155189.33 | 4557447.38 |
| **DG 38:4** | 193673.14 | 155899.54 | 0.00 | 0.00 | 120610.92 | 201609.94 | 0.00 | 0.00 | 234081.33 | 213935.29 |
| **DG 38:4\|DG 18:2_20:2** | 219973.84 | 193243.92 | 0.00 | 0.00 | 0.00 | 0.00 | 0.00 | 0.00 | 280072.56 | 295569.71 |

| Name | ET SAMPLE 1 | ET SAMPLE 2 | ET20 SAMPLE 1 | ET20 SAMPLE 2 | WSBU SAMPLE 1 | WSBU SAMPLE 2 | CH-ME SAMPLE 1 | CH-ME SAMPLE 2 | MTBE-ME SAMPLE 1 | MTBE-ME SAMPLE 2 |
|---|---|---|---|---|---|---|---|---|---|---|
| DG 39:7 | 0.00 | 0.00 | 0.00 | 0.00 | 0.00 | 0.00 | 0.00 | 0.00 | 367845.56 | 408494.13 |
| DG 39:8 | 0.00 | 0.00 | 0.00 | 0.00 | 0.00 | 0.00 | 0.00 | 0.00 | 323027.22 | 362869.42 |
| DG 40:1 | 0.00 | 0.00 | 518161.88 | 493115.08 | 0.00 | 0.00 | 0.00 | 0.00 | 0.00 | 0.00 |
| DG 40:1\|DG 22:0_18:1 | 383473.84 | 286827.37 | 300330.94 | 294485.86 | 269709.02 | 334579.67 | 0.00 | 0.00 | 319136.67 | 267860.78 |
| DG 40:2 | 9284488.65 | 9100838.76 | 7711580.11 | 8431933.70 | 9535544.97 | 9183750.72 | 8330402.64 | 5597614.61 | 10042016.89 | 8054034.95 |
| DG 40:2\|DG 22:0_18:2 | 1054434.92 | 811575.67 | 783515.41 | 836654.31 | 728500.12 | 937756.69 | 0.00 | 0.00 | 1006396.22 | 782994.47 |
| DG 40:7 | 0.00 | 0.00 | 0.00 | 0.00 | 0.00 | 0.00 | 0.00 | 0.00 | 111709.33 | 170730.00 |
| DG 42:1\|DG 24:0_18:1 | 700815.41 | 584815.15 | 511802.98 | 572451.71 | 522174.91 | 706612.43 | 0.00 | 0.00 | 570469.33 | 472430.58 |
| DG 42:2\|DG 24:0_18:2 | 1582063.46 | 1244088.97 | 1296671.16 | 1220967.96 | 1138408.67 | 1553642.54 | 0.00 | 0.00 | 1480836.56 | 1062017.57 |
| DG 42:6\|DG 16:0_26:6 | 0.00 | 0.00 | 0.00 | 0.00 | 345402.89 | 331510.28 | 0.00 | 0.00 | 0.00 | 0.00 |
| DG 44:2\|DG 26:0_18:2 | 543050.49 | 423436.96 | 461716.63 | 325635.03 | 331485.78 | 482050.11 | 0.00 | 0.00 | 379722.89 | 348787.18 |
| DG 47:1\|DG 15:0_32:1 | 0.00 | 0.00 | 0.00 | 0.00 | 0.00 | 0.00 | 106649.25 | 82950.47 | 0.00 | 0.00 |
| DG 48:1\|DG 16:0_32:1 | 0.00 | 0.00 | 0.00 | 0.00 | 147561.33 | 83104.03 | 0.00 | 0.00 | 0.00 | 0.00 |
| DG 48:2\|DG 16:0_32:2 | 0.00 | 0.00 | 0.00 | 0.00 | 0.00 | 0.00 | 111358.30 | 43190.16 | 0.00 | 0.00 |
| DG 52:1\|DG 16:0_36:1 | 182885.03 | 169334.54 | 0.00 | 0.00 | 0.00 | 0.00 | 0.00 | 0.00 | 0.00 | 0.00 |
| DG O-37:2\|DG O-21:1_16:1 | 371706.27 | 315228.71 | 324016.41 | 314061.88 | 344842.08 | 355273.92 | 909808.77 | 513841.88 | 176557.06 | 314644.17 |
| TG 24:0\|TG 8:0_8:0_8:0 | 0.00 | 0.00 | 0.00 | 0.00 | 0.00 | 0.00 | 0.00 | 0.00 | 0.00 | 0.00 |
| TG 26:0\|TG 8:0_8:0_10:0 | 0.00 | 0.00 | 0.00 | 0.00 | 0.00 | 0.00 | 114752.36 | 510826.72 | 0.00 | 0.00 |
| TG 34:0\|TG 10:0_12:0_12:0 | 476923.30 | 483353.61 | 520450.55 | 441533.87 | 0.00 | 0.00 | 0.00 | 0.00 | 0.00 | 0.00 |
| TG 34:0\|TG 8:0_12:0_14:0 | 258098.05 | 242076.91 | 0.00 | 0.00 | 378263.41 | 386870.19 | 511376.93 | 438694.84 | 679574.33 | 601357.18 |
| TG 36:0\|TG 10:0_12:0_14:0 | 306525.84 | 336427.53 | 0.00 | 0.00 | 236535.49 | 340524.42 | 417140.19 | 543616.80 | 461889.06 | 172243.88 |
| TG 36:0\|TG 12:0_12:0_12:0 | 408452.43 | 476688.09 | 477137.68 | 368631.88 | 484706.24 | 565596.13 | 1053517.64 | 853541.64 | 485888.72 | 495815.19 |
| TG 36:0\|TG 8:0_12:0_16:0 | 0.00 | 0.00 | 0.00 | 0.00 | 0.00 | 0.00 | 0.00 | 0.00 | 0.00 | 0.00 |
| TG 38:0\|TG 10:0_12:0_16:0 | 0.00 | 0.00 | 0.00 | 0.00 | 428478.12 | 374162.82 | 657412.83 | 559517.81 | 0.00 | 0.00 |
| TG 38:0\|TG 12:0_12:0_14:0 | 181112.59 | 301831.96 | 383115.25 | 458523.98 | 0.00 | 0.00 | 450515.85 | 558865.00 | 350724.78 | 287593.69 |
| TG 38:0\|TG 8:0_12:0_18:0 | 0.00 | 0.00 | 0.00 | 0.00 | 0.00 | 0.00 | 0.00 | 0.00 | 0.00 | 0.00 |
| TG 40:0\|TG 10:0_14:0_16:0 | 627583.57 | 546724.79 | 664397.96 | 483762.21 | 670729.77 | 940716.02 | 1502131.23 | 1247911.64 | 872852.22 | 543481.26 |
| TG 40:0\|TG 12:0_12:0_16:0 | 0.00 | 0.00 | 0.00 | 0.00 | 0.00 | 0.00 | 0.00 | 0.00 | 0.00 | 0.00 |
| TG 40:1\|TG 8:0_14:0_18:1 | 0.00 | 0.00 | 0.00 | 0.00 | 0.00 | 0.00 | 0.00 | 0.00 | 0.00 | 0.00 |
| TG 41:0\|TG 12:0_14:0_15:0 | 238842.16 | 442684.33 | 0.00 | 0.00 | 225271.50 | 506014.59 | 736994.53 | 718185.86 | 655562.50 | 345360.68 |
| TG 42:0\|TG 12:0_14:0_16:0 | 2732497.08 | 2851335.26 | 3163409.72 | 3050492.43 | 2431152.60 | 2689531.49 | 5702904.15 | 4425188.44 | 2941414.22 | 2314514.37 |
| TG 42:0\|TG 14:0_14:0_14:0 | 314804.16 | 309653.09 | 0.00 | 0.00 | 432620.23 | 296907.24 | 1352717.36 | 1036167.42 | 354715.11 | 218304.85 |
| TG 42:1\|TG 12:0_14:0_16:1 | 576696.38 | 427138.76 | 629405.25 | 299253.59 | 528843.24 | 583204.25 | 1550293.96 | 1430875.00 | 1185009.89 | 741466.46 |
| TG 43:0\|TG 12:0_15:0_16:0 | 0.00 | 0.00 | 0.00 | 0.00 | 26631.68 | 7876.63 | 0.00 | 0.00 | 0.00 | 0.00 |
| TG 43:0\|TG 13:0_14:0_16:0 | 3083995.03 | 3375363.09 | 3897486.41 | 3507739.28 | 3677792.14 | 3403501.22 | 8442777.74 | 6477100.27 | 3384907.33 | 3086429.32 |
| TG 43:1\|TG 13:0_14:0_16:1 | 0.00 | 0.00 | 0.00 | 0.00 | 282342.20 | 292789.28 | 896855.47 | 204602.81 | 1775819.00 | 1038766.31 |
| TG 43:1\|TG 14:0_15:0_14:1 | 1792903.68 | 1617458.25 | 1830639.78 | 1672780.66 | 1811757.34 | 1706635.91 | 3890875.47 | 3031272.81 | 0.00 | 0.00 |
| TG 44:0\|TG 13:0_15:0_16:0 | 5595680.87 | 6199946.80 | 5975295.03 | 5794422.54 | 7462925.67 | 6313236.24 | 19375297.55 | 13169646.88 | 6606694.67 | 5726427.18 |
| TG 44:0\|TG 14:0_15:0_15:0 | 377930.86 | 402933.71 | 408907.07 | 438714.03 | 0.00 | 0.00 | 0.00 | 0.00 | 533119.28 | 426720.00 |
| TG 44:1\|TG 12:0_16:0_16:1 | 4262973.41 | 4242059.38 | 0.00 | 0.00 | 4759680.46 | 4276497.68 | 9143950.94 | 6636005.00 | 4693645.33 | 4117186.80 |
| TG 44:1\|TG 13:0_15:0_16:1 | 0.00 | 0.00 | 0.00 | 0.00 | 0.00 | 0.00 | 0.00 | 0.00 | 654128.33 | 544860.49 |
| TG 44:1\|TG 14:0_14:0_16:1 | 610683.41 | 546104.95 | 519265.58 | 583682.65 | 0.00 | 0.00 | 2358122.45 | 1793598.91 | 0.00 | 0.00 |
| TG 44:1\|TG 14:0_16:0_14:1 | 0.00 | 0.00 | 4449301.66 | 4323253.92 | 0.00 | 0.00 | 0.00 | 0.00 | 0.00 | 0.00 |
| TG 44:2\|TG 10:0_16:1_18:1 | 381597.84 | 393358.87 | 424114.92 | 174577.62 | 366771.45 | 365544.97 | 0.00 | 0.00 | 0.00 | 0.00 |
| TG 44:2\|TG 14:0_14:1_16:1 | 1111503.68 | 241296.29 | 499581.27 | 997550.83 | 617648.67 | 1089827.40 | 2619486.98 | 2066619.69 | 1137315.89 | 601775.19 |
| TG 45:0\|TG 14:0_15:0_16:0 | 7938977.68 | 8331730.36 | 7700086.96 | 8350007.51 | 5289773.64 | 3909406.69 | 22230555.85 | 17418542.03 | 9128622.56 | 7072383.20 |
| TG 45:1\|TG 13:0_16:0_16:1 | 0.00 | 0.00 | 517710.94 | 525469.01 | 0.00 | 0.00 | 0.00 | 0.00 | 0.00 | 0.00 |
| TG 45:1\|TG 14:0_15:0_16:1 | 6749147.62 | 6322386.44 | 5971180.11 | 6622813.70 | 2803022.06 | 2339211.18 | 17966838.49 | 12695347.66 | 6634361.33 | 5628031.07 |
| TG 45:2\|TG 14:0_15:1_16:1 | 1584647.35 | 2445008.25 | 0.00 | 0.00 | 2546536.19 | 1893520.00 | 4449829.43 | 3734080.00 | 2544142.89 | 2270560.97 |
| TG 45:2\|TG 15:0_14:1_16:1 | 0.00 | 0.00 | 2051339.45 | 1819444.20 | 0.00 | 0.00 | 0.00 | 0.00 | 0.00 | 0.00 |
| TG 46:0\|TG 14:0_15:0_17:0 | 223587.03 | 251824.54 | 0.00 | 0.00 | 945007.63 | 2012358.23 | 3302075.47 | 2048915.47 | 308874.56 | 285310.19 |
| TG 46:0\|TG 14:0_16:0_16:0 | 10503436.11 | 9187533.20 | 8673575.53 | 10315890.28 | 13420034.22 | 6274782.43 | 27932368.30 | 20485501.25 | 10087704.00 | 9332438.84 |
| TG 46:0\|TG 15:0_15:0_16:0 | 0.00 | 0.00 | 0.00 | 0.00 | 0.00 | 0.00 | 0.00 | 0.00 | 0.00 | 0.00 |
| TG 46:1\|TG 14:0_16:0_16:1 | 9526800.54 | 9919075.98 | 10277050.06 | 9785152.71 | 11734818.70 | 9364200.14 | 27618669.81 | 21189051.25 | 11474760.94 | 8740409.76 |
| TG 46:2\|TG 12:0_16:1_18:1 | 510093.62 | 506537.42 | 0.00 | 0.00 | 0.00 | 0.00 | 0.00 | 0.00 | 0.00 | 0.00 |
| TG 46:2\|TG 14:0_16:1_16:1 | 0.00 | 0.00 | 513042.76 | 477195.80 | 0.00 | 0.00 | 2362470.76 | 1752704.84 | 460601.83 | 449032.43 |
| TG 46:2\|TG 16:0_14:1_16:1 | 5695942.05 | 5463205.36 | 5507214.14 | 5188767.29 | 6715967.63 | 5930435.36 | 9962576.60 | 7478396.88 | 5953580.00 | 5119974.76 |
| TG 46:2\|TG 16:0_15:1_15:1 | 0.00 | 0.00 | 0.00 | 0.00 | 563386.19 | 353275.30 | 0.00 | 0.00 | 0.00 | 0.00 |
| TG 46:3\|TG 10:0_18:1_18:2 | 0.00 | 0.00 | 165605.41 | 133755.30 | 142483.58 | 178292.27 | 0.00 | 0.00 | 158660.28 | 150939.81 |
| TG 46:3\|TG 14:1_16:1_16:1 | 1026986.16 | 360113.04 | 888047.24 | 748359.34 | 820868.50 | 950961.33 | 1608833.96 | 1321820.78 | 887646.61 | 352701.07 |
| TG 46:4\|TG 10:0_18:2_18:2 | 0.00 | 0.00 | 142522.65 | 111996.52 | 0.00 | 0.00 | 0.00 | 0.00 | 0.00 | 0.00 |
| TG 47:0\|TG 14:0_16:0_17:0 | 12274979.46 | 10998912.99 | 303121.99 | 292589.01 | 409376.47 | 225143.65 | 4410635.85 | 2425984.06 | 13625538.67 | 11684096.50 |
| TG 47:0\|TG 15:0_16:0_16:0 | 303657.24 | 220336.65 | 8730306.30 | 12765009.50 | 12798761.62 | 11983570.39 | 30062541.89 | 21695710.00 | 0.00 | 0.00 |
| TG 47:1\|TG 14:0_15:0_18:1 | 0.00 | 0.00 | 0.00 | 0.00 | 0.00 | 0.00 | 0.00 | 0.00 | 698680.56 | 582977.67 |
| TG 47:1\|TG 15:0_16:0_16:1 | 12352425.89 | 10915375.67 | 10059114.75 | 11661359.72 | 14955505.03 | 9321977.24 | 33177564.15 | 24397492.34 | 13523736.00 | 10157728.93 |
| TG 47:2\|TG 15:0_15:1_17:1 | 0.00 | 0.00 | 0.00 | 0.00 | 722767.92 | 545028.90 | 0.00 | 0.00 | 0.00 | 0.00 |
| TG 47:2\|TG 15:0_16:1_16:1 | 0.00 | 0.00 | 6032620.55 | 6049056.80 | 0.00 | 0.00 | 2747338.87 | 1946293.59 | 664889.50 | 529915.19 |
| TG 47:2\|TG 16:0_15:1_16:1 | 6410787.95 | 6757572.27 | 589781.49 | 593831.05 | 7104301.97 | 6153607.07 | 12566086.79 | 10250346.88 | 7115113.78 | 5761086.60 |
| TG 47:3\|TG 14:1_16:1_17:1 | 752107.24 | 944139.28 | 1257153.70 | 1219496.24 | 1323230.75 | 1340274.37 | 0.00 | 0.00 | 1271303.33 | 1153236.31 |
| TG 47:3\|TG 15:1_16:1_16:1 | 0.00 | 0.00 | 0.00 | 0.00 | 0.00 | 0.00 | 2839211.98 | 2213671.88 | 0.00 | 0.00 |
| TG 48:0\|TG 15:0_16:0_17:0 | 26074773.62 | 19876753.92 | 15109550.50 | 18044145.80 | 18407219.42 | 14804222.76 | 38471362.64 | 25636671.56 | 17502369.33 | 14588221.65 |
| TG 48:0\|TG 16:0_16:0_16:0 | 0.00 | 0.00 | 0.00 | 0.00 | 48401.39 | 28681.38 | 0.00 | 0.00 | 0.00 | 0.00 |
| TG 48:1\|TG 14:0_16:0_18:1 | 16397685.62 | 16186444.54 | 14894219.67 | 17043763.98 | 8099495.18 | 11863248.51 | 5203490.09 | 3424394.38 | 20414005.33 | 16697691.65 |
| TG 48:1\|TG 15:0_17:0_16:1 | 209036.54 | 243654.64 | 0.00 | 0.00 | 0.00 | 0.00 | 0.00 | 0.00 | 335821.39 | 204020.68 |
| TG 48:1\|TG 16:0_16:0_16:1 | 0.00 | 0.00 | 129978.34 | 264698.45 | 0.00 | 0.00 | 40209107.92 | 30611395.00 | 0.00 | 0.00 |
| TG 48:2\|TG 14:0_16:0_18:2 | 17234338.59 | 16428450.31 | 17263414.81 | 15880387.18 | 16812742.66 | 17393958.90 | 0.00 | 0.00 | 445657.17 | 446411.17 |
| TG 48:2\|TG 14:0_16:1_18:1 | 513106.32 | 533932.58 | 558034.59 | 570319.34 | 481798.58 | 353112.18 | 3404027.93 | 2410163.28 | 695789.61 | 1423459.90 |
| TG 48:2\|TG 16:0_16:1_16:1 | 0.00 | 0.00 | 0.00 | 0.00 | 118911.85 | 31632.54 | 3404027.93 | 2410163.28 | 17975771.44 | 13888293.88 |
| TG 48:3\|TG 12:0_18:1_18:2 | 0.00 | 0.00 | 0.00 | 0.00 | 189709.71 | 270349.06 | 0.00 | 0.00 | 0.00 | 0.00 |
| TG 48:3\|TG 14:1_16:1_18:1 | 5686126.97 | 5367391.96 | 5405120.44 | 4583253.48 | 4841583.82 | 5782792.04 | 6359349.43 | 4822581.88 | 4874976.89 | 4449006.60 |
| TG 48:3\|TG 16:1_16:1_16:1 | 0.00 | 0.00 | 191807.40 | 262445.36 | 319826.24 | 18366.24 | 1195570.09 | 1065070.23 | 0.00 | 0.00 |
| TG 48:4\|TG 12:0_18:2_18:2 | 660241.46 | 574905.31 | 614589.72 | 498741.33 | 467602.08 | 658778.51 | 0.00 | 0.00 | 371541.11 | 335882.82 |
| TG 48:4\|TG 14:1_16:1_18:2 | 682301.41 | 685675.41 | 756409.39 | 582991.55 | 529172.43 | 733030.50 | 0.00 | 0.00 | 592691.61 | 499693.30 |

| Name | ET SAMPLE 1 | ET SAMPLE 2 | ET20 SAMPLE 1 | ET20 SAMPLE 2 | WSBU SAMPLE 1 | WSBU SAMPLE 2 | CH-ME SAMPLE 1 | CH-ME SAMPLE 2 | MTBE-ME SAMPLE 1 | MTBE-ME SAMPLE 2 |
|---|---|---|---|---|---|---|---|---|---|---|
| TG 49:0\|TG 15:0_16:0_18:0 | 8091151.57 | 6973835.05 | 7682243.54 | 8923196.46 | 8472427.51 | 8293708.95 | 20934272.45 | 17749996.41 | 8525117.33 | 7837065.39 |
| TG 49:0\|TG 16:0_16:0_17:0 | 243657.51 | 268450.00 | 344563.31 | 259447.18 | 0.00 | 0.00 | 0.00 | 0.00 | 0.00 | 0.00 |
| TG 49:1\|TG 15:0_16:0_18:1 | 13047474.16 | 10825797.11 | 12258301.88 | 13272690.39 | 14028113.76 | 11798255.03 | 32983165.85 | 23934617.03 | 13067202.89 | 12725055.24 |
| TG 49:1\|TG 16:0_17:0_16:1 | 371445.62 | 297894.64 | 274516.91 | 384151.49 | 476507.05 | 331878.67 | 0.00 | 0.00 | 0.00 | 0.00 |
| TG 49:2\|TG 15:0_16:0_18:2 | 0.00 | 0.00 | 0.00 | 0.00 | 0.00 | 0.00 | 2462371.89 | 1784479.22 | 522363.56 | 452050.73 |
| TG 49:2\|TG 15:0_16:1_18:1 | 10096646.92 | 9555214.85 | 9990533.76 | 10003924.36 | 11150346.88 | 8428812.38 | 0.00 | 0.00 | 10807329.78 | 8610569.32 |
| TG 49:2\|TG 16:0_16:1_17:1 | 552004.05 | 455610.98 | 0.00 | 0.00 | 0.00 | 0.00 | 22208073.96 | 17091092.50 | 0.00 | 0.00 |
| TG 49:3\|TG 15:1_16:1_18:1 | 3679424.87 | 3658462.68 | 349983.87 | 380961.33 | 3813015.26 | 4099373.70 | 5992068.30 | 4067779.06 | 3634483.11 | 2883345.83 |
| TG 49:3\|TG 16:0_15:1_18:2 | 364983.57 | 360510.52 | 3210726.41 | 3379366.19 | 385521.73 | 401403.43 | 0.00 | 0.00 | 377939.22 | 296489.27 |
| TG 49:3\|TG 16:1_16:1_17:1 | 0.00 | 0.00 | 0.00 | 0.00 | 0.00 | 0.00 | 1069308.68 | 736460.08 | 0.00 | 0.00 |
| TG 50:0\|TG 15:0_17:0_18:0 | 0.00 | 0.00 | 0.00 | 0.00 | 0.00 | 0.00 | 3876713.21 | 2963178.13 | 0.00 | 0.00 |
| TG 50:0\|TG 16:0_16:0_18:0 | 8918436.97 | 8474419.79 | 8253435.36 | 7829914.81 | 8536002.20 | 8728344.39 | 16028559.15 | 10292856.56 | 8558143.78 | 7583529.90 |
| TG 50:1\|TG 16:0_16:0_18:1 | 109113837.00 | 97685191.75 | 105999700.80 | 92976405.97 | 85838898.55 | 124665878.50 | 38332646.04 | 35020817.50 | 85096993.11 | 89475047.18 |
| TG 50:2\|TG 16:0_16:0_18:2 | 186216757.90 | 158094892.60 | 161455446.40 | 139801762.00 | 145720911.50 | 199027666.90 | 0.00 | 0.00 | 144973264.70 | 135175851.10 |
| TG 50:2\|TG 16:0_16:1_18:1 | 4393195.68 | 3723434.23 | 3912046.85 | 3714403.98 | 3990303.82 | 3973418.34 | 44410372.08 | 40492550.31 | 0.00 | 0.00 |
| TG 50:3\|TG 14:0_18:1_18:2 | 35513502.16 | 33421565.05 | 34465636.80 | 30058031.16 | 878974.80 | 1187867.96 | 0.00 | 0.00 | 26287916.44 | 24718732.43 |
| TG 50:3\|TG 16:0_16:1_18:2 | 0.00 | 0.00 | 0.00 | 0.00 | 14992328.01 | 19907108.70 | 0.00 | 0.00 | 1370672.56 | 736204.32 |
| TG 50:4\|TG 14:0_18:2_18:2 | 18293575.57 | 16479420.21 | 18753253.48 | 14544988.51 | 12916846.24 | 19796779.34 | 917946.13 | 1307120.55 | 12460538.78 | 13467420.00 |
| TG 50:5\|TG 14:0_18:2_18:3 | 3274980.54 | 2714077.01 | 3265474.20 | 2411615.91 | 2174964.16 | 3580760.72 | 0.00 | 0.00 | 2314261.11 | 2003742.43 |
| TG 50:6\|TG 14:1_18:2_18:3 | 392225.68 | 328639.64 | 396768.62 | 310605.08 | 280214.45 | 405846.19 | 0.00 | 0.00 | 327766.56 | 250961.41 |
| TG 51:0\|TG 16:0_17:0_18:0 | 3300728.00 | 2919164.95 | 2997644.86 | 3313537.24 | 3771135.03 | 3240302.98 | 5610157.74 | 3867119.84 | 3558473.78 | 2947202.33 |
| TG 51:0\|TG 17:0_17:0_17:0 | 0.00 | 0.00 | 0.00 | 0.00 | 0.00 | 0.00 | 0.00 | 0.00 | 0.00 | 0.00 |
| TG 51:1\|TG 15:0_18:0_18:1 | 233684.65 | 162104.95 | 0.00 | 0.00 | 0.00 | 0.00 | 0.00 | 0.00 | 0.00 | 0.00 |
| TG 51:1\|TG 16:0_17:0_18:1 | 6847473.73 | 5728490.72 | 6609205.97 | 6458247.07 | 6436545.67 | 7116194.92 | 14562724.06 | 10598702.27 | 7059502.00 | 5894429.81 |
| TG 51:1\|TG 17:0_18:0_16:1 | 0.00 | 0.00 | 0.00 | 0.00 | 286979.08 | 215056.57 | 0.00 | 0.00 | 0.00 | 0.00 |
| TG 51:2\|TG 16:0_17:1_18:1 | 8790845.03 | 7568817.06 | 8364288.18 | 8047417.46 | 286654.68 | 276483.43 | 1289106.42 | 914004.14 | 7871317.33 | 8211996.07 |
| TG 51:2\|TG 17:0_16:1_18:1 | 0.00 | 0.00 | 0.00 | 0.00 | 8863516.30 | 8469212.82 | 0.00 | 0.00 | 0.00 | 0.00 |
| TG 51:3\|TG 15:0_18:1_18:2 | 5869750.49 | 5492206.60 | 4783889.50 | 4944220.99 | 5084006.94 | 6243000.66 | 0.00 | 0.00 | 4528889.33 | 4588041.17 |
| TG 51:3\|TG 15:1_18:1_18:1 | 0.00 | 0.00 | 251165.64 | 245251.38 | 0.00 | 0.00 | 0.00 | 0.00 | 0.00 | 0.00 |
| TG 51:3\|TG 16:1_17:1_18:1 | 322595.41 | 276166.80 | 0.00 | 0.00 | 1734840.69 | 1653826.41 | 0.00 | 0.00 | 227184.67 | 257792.62 |
| TG 51:4\|TG 15:0_18:2_18:2 | 3457653.19 | 3459860.21 | 3681022.76 | 2844971.71 | 2615802.08 | 4336971.49 | 0.00 | 0.00 | 2745102.44 | 2475383.50 |
| TG 51:4\|TG 15:1_18:1_18:2 | 430418.81 | 414745.98 | 484924.20 | 377050.72 | 887376.24 | 1087360.22 | 1411750.47 | 1118379.14 | 338788.56 | 298431.46 |
| TG 51:5\|TG 15:1_18:2_18:2 | 1809434.70 | 1681279.59 | 1924136.80 | 1580305.30 | 1219566.53 | 2032287.07 | 1382407.74 | 1205699.69 | 1332010.44 | 1319145.83 |
| TG 51:6\|TG 15:1_18:2_18:3 | 115983.30 | 69772.58 | 110308.73 | 74981.38 | 60306.30 | 127592.98 | 0.00 | 0.00 | 0.00 | 0.00 |
| TG 52:0\|TG 12:0_12:0_28:0 | 0.00 | 0.00 | 0.00 | 0.00 | 0.00 | 0.00 | 0.00 | 0.00 | 0.00 | 0.00 |
| TG 52:0\|TG 14:0_18:0_20:0 | 3286857.30 | 2995409.28 | 3303512.04 | 3035691.05 | 3506217.80 | 3457152.04 | 7309692.83 | 5071081.25 | 3399528.44 | 3149790.29 |
| TG 52:0\|TG 16:0_18:0_18:0 | 806037.08 | 722391.34 | 768906.30 | 686394.20 | 623667.40 | 1130278.23 | 805354.81 | 504900.78 | 662195.61 | 675458.59 |
| TG 52:1\|TG 16:0_18:0_18:1 | 31416844.11 | 27315551.55 | 27290772.60 | 21998427.51 | 23754607.63 | 40992370.06 | 16715682.08 | 11849486.88 | 21772858.22 | 25092124.47 |
| TG 52:1\|TG 18:0_18:0_16:1 | 2127250.81 | 1433693.51 | 0.00 | 0.00 | 1367595.38 | 2217628.07 | 0.00 | 0.00 | 0.00 | 0.00 |
| TG 52:2\|TG 16:0_18:1_18:1 | 392203059.00 | 348911751.80 | 370594179.90 | 322205391.40 | 296939089.90 | 465409332.20 | 53485782.26 | 59250508.44 | 285449928.90 | 318098771.30 |
| TG 52:2\|TG 18:0_16:1_18:1 | 2574307.89 | 3029228.66 | 2924920.44 | 2623664.53 | 2602049.25 | 3707219.45 | 0.00 | 0.00 | 2551723.33 | 1988606.41 |
| TG 52:3\|TG 16:0_18:1_18:2 | 631149734.90 | 547935239.60 | 559266647.50 | 487732400.40 | 485161411.30 | 694523527.10 | | | 488978040.00 | 471197352.20 |
| TG 52:3\|TG 16:1_18:1_18:1 | 0.00 | 0.00 | 0.00 | 0.00 | 845327.51 | 1820403.09 | 54787631.32 | 68019373.44 | 0.00 | 0.00 |
| TG 52:3\|TG 17:1_17:1_18:1 | 1645642.38 | 1538915.46 | 0.00 | 0.00 | 0.00 | 0.00 | 0.00 | 0.00 | 0.00 | 0.00 |
| TG 52:4\|TG 16:0_18:1_18:3 | 15329458.16 | 14928831.34 | 14270637.79 | 14020709.83 | 14021009.02 | 16508301.44 | 0.00 | 0.00 | 14804856.89 | 11575019.03 |
| TG 52:4\|TG 16:0_18:2_18:2 | 470076609.70 | 436545187.60 | 450901653.90 | 378965993.40 | 364772949.80 | 520491788.70 | 31959365.28 | 44713409.69 | 361092323.60 | 329551161.20 |
| TG 52:5\|TG 16:0_18:2_18:3 | 63194986.38 | 66270617.73 | 76122513.59 | 56858247.96 | 50946965.55 | 82979462.10 | 3475560.76 | 4827188.83 | 49483210.22 | 50087716.89 |
| TG 52:6\|TG 16:0_18:3_18:3 | 3143204.97 | 2782974.59 | 2809100.11 | 1992176.58 | 2186415.03 | 3260615.47 | 0.00 | 0.00 | 1799135.56 | 1616505.53 |
| TG 52:6\|TG 16:1_18:2_18:3 | 0.00 | 0.00 | 632399.56 | 461660.99 | 0.00 | 0.00 | 0.00 | 0.00 | 0.00 | 0.00 |
| TG 53:0\|TG 14:0_15:0_24:0 | 2462479.14 | 2876142.89 | 2607100.77 | 2414731.05 | 2519238.61 | 3224355.14 | 5429653.96 | 3814991.88 | 0.00 | 0.00 |
| TG 53:0\|TG 17:0_17:0_19:0 | 0.00 | 0.00 | 0.00 | 0.00 | 0.00 | 0.00 | 1904409.81 | 1025333.13 | 0.00 | 0.00 |
| TG 53:0\|TG 14:0_16:0_23:0 | 0.00 | 0.00 | 0.00 | 0.00 | 0.00 | 0.00 | 0.00 | 0.00 | 2819919.56 | 2953059.22 |
| TG 53:1\|TG 17:0_18:0_18:1 | 2810884.97 | 2690709.90 | 0.00 | 0.00 | 2991888.79 | 3004857.90 | 6750393.21 | 4155863.13 | 3046230.22 | 2619062.91 |
| TG 53:1\|TG 17:0_20:0_16:1 | 0.00 | 0.00 | 2905964.20 | 2776596.24 | 0.00 | 0.00 | 0.00 | 0.00 | 0.00 | 0.00 |
| TG 53:2\|TG 17:0_18:1_18:1 | 4853187.46 | 3869657.32 | 4498773.04 | 4071291.49 | 3944736.65 | 5058997.57 | 3090153.02 | 2527169.38 | 3814962.67 | 3882298.64 |
| TG 53:2\|TG 18:0_17:1_18:1 | 4040598.05 | 3718634.64 | 4095071.82 | 3833121.33 | 0.00 | 0.00 | 6197583.02 | 4056625.00 | 0.00 | 0.00 |
| TG 53:3\|TG 17:0_18:1_18:2 | 5199651.46 | 4350412.78 | 4998643.54 | 4315512.49 | 4328311.68 | 6019799.34 | 4198486.42 | 3190658.13 | 4199853.78 | 3963404.27 |
| TG 53:4\|TG 17:1_18:1_18:2 | 3575751.35 | 3526931.55 | 3553996.91 | 3130023.65 | 1600528.30 | 2304449.12 | 1625992.83 | 1367553.44 | 2688926.44 | 2555038.64 |
| TG 53:5\|TG 17:1_18:2_18:2 | 164388.54 | 167332.94 | 1535923.65 | 1318462.21 | 1262570.06 | 1613098.79 | 332789.81 | 255302.42 | 0.00 | 0.00 |
| TG 53:5\|TG 18:1_17:2_18:2 | 1360585.73 | 1275894.95 | 175013.54 | 110028.23 | 124086.65 | 203516.85 | 0.00 | 0.00 | 824111.89 | 1022932.43 |
| TG 53:6\|TG 17:2_18:2_18:2 | 0.00 | 0.00 | 0.00 | 0.00 | 169113.24 | 275524.20 | 0.00 | 0.00 | 0.00 | 0.00 |
| TG 54:0\|TG 14:0_16:0_24:0 | 2976292.97 | 2644034.85 | 2756183.20 | 2846105.41 | 0.00 | 0.00 | 8893715.09 | 6404299.06 | 3173205.56 | 3061359.03 |
| TG 54:0\|TG 15:0_16:0_23:0 | 0.00 | 0.00 | 0.00 | 0.00 | 3020728.56 | 2876436.02 | 0.00 | 0.00 | 0.00 | 0.00 |
| TG 54:0\|TG 18:0_18:0_18:0 | 0.00 | 0.00 | 199108.62 | 209862.71 | 0.00 | 0.00 | 0.00 | 0.00 | 0.00 | 0.00 |
| TG 54:1\|TG 16:0_20:0_18:1 | 13383727.89 | 12750724.69 | 12266364.14 | 9690378.45 | 10338193.12 | 18044672.49 | 0.00 | 0.00 | 10356037.17 | 11561134.13 |
| TG 54:1\|TG 18:0_18:0_18:1 | 0.00 | 0.00 | 0.00 | 0.00 | 0.00 | 0.00 | 11461938.49 | 7974027.81 | 0.00 | 0.00 |
| TG 54:2\|TG 16:0_18:1_20:1 | 3498756.11 | 3192587.63 | 3136169.28 | 2706072.71 | 2845762.31 | 4089567.29 | 1175515.66 | 960227.81 | 2472640.22 | 2862720.78 |
| TG 54:2\|TG 18:0_18:1_18:1 | 68574872.22 | 61189087.01 | 60214870.28 | 43117362.21 | 46236922.08 | 95660828.29 | 13193299.62 | 10366606.88 | 41515246.22 | 52136164.66 |
| TG 54:3\|TG 18:1_18:1_18:1 | 456285016.20 | 408384216.90 | 421305981.90 | 377108760.20 | 184100293.60 | 289923110.60 | 54769281.89 | 61645404.38 | 331426108.40 | 382305804.30 |
| TG 54:4\|TG 18:1_18:1_18:2 | 637825174.50 | 566972240.80 | 558045626.50 | 489705659.20 | 495912565.10 | 732843486.40 | 48794972.45 | 64257770.94 | 490857579.60 | 493933070.30 |
| TG 54:5\|TG 18:1_18:2_18:2 | 748374114.60 | 708851640.40 | 691646186.10 | 604602197.60 | 596595527.40 | 850028400.40 | 47940110.57 | 68075898.13 | 568577838.20 | 526368118.10 |
| TG 54:6\|TG 18:1_18:2_18:3 | 413461661.40 | 409990560.00 | 454422978.10 | 352289128.00 | 324460659.40 | 500274362.00 | 19189868.68 | 26214227.50 | 294914730.70 | 294256714.60 |
| TG 54:6\|TG 18:2_18:2_18:2 | 1287001.95 | 270230.82 | 0.00 | 0.00 | 0.00 | 0.00 | 3933956.23 | 5277636.25 | 21940387.56 | 20174940.58 |
| TG 54:7\|TG 18:2_18:2_18:3 | 52742734.70 | 49458743.92 | 55128088.40 | 39892017.24 | 36247961.16 | 56917349.83 | 1833657.45 | 3169557.89 | 34206233.33 | 33391630.68 |
| TG 54:8\|TG 18:2_18:3_18:3 | 2839328.27 | 2447862.06 | 3116543.48 | 2277348.01 | 1712979.19 | 3091406.35 | 0.00 | 0.00 | 2056075.44 | 1840531.46 |

| Name | ET SAMPLE 1 | ET SAMPLE 2 | ET20 SAMPLE 1 | ET20 SAMPLE 2 | WSBU SAMPLE 1 | WSBU SAMPLE 2 | CH-ME SAMPLE 1 | CH-ME SAMPLE 2 | MTBE-ME SAMPLE 1 | MTBE-ME SAMPLE 2 |
|---|---|---|---|---|---|---|---|---|---|---|
| TG 55:0\|TG 15:0_16:0_24:0 | 3235312.43 | 2927721.65 | 3461941.27 | 3451249.61 | 3686346.01 | 3532558.23 | 8212551.13 | 5691164.53 | 3594344.00 | 3265711.46 |
| TG 55:1\|TG 15:0_24:0_16:1 | 2700939.03 | 2427777.11 | 2733556.24 | 2656408.18 | 2604900.81 | 2962990.94 | 5427485.66 | 4002557.81 | 2672888.44 | 2558289.32 |
| TG 55:2\|TG 19:0_18:1_18:1 | 0.00 | 0.00 | 8430544.97 | 8436851.27 | 0.00 | 0.00 | 3534957.74 | 3372023.75 | 7462042.67 | 6945140.97 |
| TG 55:2\|TG 21:0_16:1_18:1 | 2246687.35 | 2081707.42 | 2170372.82 | 1964220.33 | 2075901.50 | 2386135.69 | 3559097.74 | 2419164.53 | 2063547.11 | 1925673.59 |
| TG 55:2\|TG 24:0_15:1_16:1 | 0.00 | 0.00 | 0.00 | 0.00 | 0.00 | 0.00 | 0.00 | 0.00 | 0.00 | 0.00 |
| TG 55:3\|TG 17:1_18:1_20:1 | 0.00 | 0.00 | 0.00 | 0.00 | 0.00 | 0.00 | 1687498.49 | 1050485.86 | 0.00 | 0.00 |
| TG 55:3\|TG 18:1_18:1_19:1 | 1792516.65 | 1807094.02 | 1834908.62 | 1666536.91 | 1580701.50 | 2380127.51 | 0.00 | 0.00 | 1352833.33 | 1602066.21 |
| TG 55:3\|TG 19:0_18:1_18:2 | 0.00 | 0.00 | 0.00 | 0.00 | 9839641.16 | 10350108.29 | 0.00 | 0.00 | 0.00 | 0.00 |
| TG 55:4\|TG 18:1_19:1_18:2 | 0.00 | 0.00 | 0.00 | 0.00 | 892966.01 | 1427960.00 | 0.00 | 0.00 | 887013.67 | 1002642.33 |
| TG 55:5\|TG 19:1_18:2_18:2 | 1141767.35 | 642376.96 | 0.00 | 0.00 | 0.00 | 0.00 | 0.00 | 0.00 | 0.00 | 0.00 |
| TG 56:0\|TG 15:0_16:0_25:0 | 3705939.89 | 3581210.72 | 3735714.92 | 3560459.67 | 3939336.42 | 3096385.41 | 7903836.98 | 5373000.63 | 3960744.00 | 3910168.93 |
| TG 56:0\|TG 16:0_16:0_24:0 | 0.00 | 0.00 | 243320.44 | 248237.13 | 293321.73 | 202590.11 | 591403.49 | 396939.92 | 0.00 | 0.00 |
| TG 56:1\|TG 15:0_25:0_16:1 | 0.00 | 0.00 | 4739923.98 | 3823998.67 | 0.00 | 0.00 | 0.00 | 0.00 | 0.00 | 0.00 |
| TG 56:1\|TG 16:0_22:0_18:1 | 358065.68 | 364745.46 | 300011.27 | 269118.18 | 4482043.93 | 7032407.07 | 0.00 | 0.00 | 368852.11 | 361736.41 |
| TG 56:1\|TG 16:0_24:0_16:1 | 5278450.60 | 4633947.22 | 0.00 | 0.00 | 327690.92 | 446526.91 | 8420520.38 | 6057517.34 | 4828251.11 | 5381638.84 |
| TG 56:2\|TG 20:0_18:1_18:1 | 22039550.92 | 20875471.03 | 19670867.18 | 13635395.86 | 14321261.62 | 33007987.18 | 0.00 | 0.00 | 12511416.89 | 17539330.49 |
| TG 56:2\|TG 22:0_16:1_18:1 | 0.00 | 0.00 | 0.00 | 0.00 | 0.00 | 0.00 | 4562418.49 | 3854465.94 | 927796.94 | 1219211.36 |
| TG 56:3\|TG 18:1_18:1_20:1 | 40871586.59 | 35735808.66 | 36606579.23 | 26171932.71 | 1590505.78 | 2675432.93 | 0.00 | 0.00 | 0.00 | 0.00 |
| TG 56:3\|TG 20:0_18:1_18:2 | 2141737.73 | 1959142.27 | 0.00 | 0.00 | 26476526.24 | 60474625.41 | 2794301.70 | 2773096.56 | 24306865.89 | 33626917.28 |
| TG 56:4\|TG 18:1_20:1_18:2 | 40291467.57 | 35035468.76 | 36397627.18 | 30176456.02 | 29363650.64 | 52732677.24 | 2273933.77 | 2799461.56 | 26657334.33 | 33702658.54 |
| TG 56:5\|TG 20:0_18:2_18:3 | 351002.27 | 318585.88 | 0.00 | 0.00 | 251056.07 | 417237.24 | 0.00 | 0.00 | 0.00 | 0.00 |
| TG 56:5\|TG 20:1_18:2_18:2 | 18018098.16 | 15731541.44 | 15118585.64 | 12668794.70 | 13608745.43 | 20951536.80 | 1890519.81 | 1996226.41 | 13642870.56 | 13396351.55 |
| TG 56:6\|TG 18:2_18:2_20:2 | 3203597.19 | 2808135.46 | 2690813.70 | 2325556.24 | 2320697.11 | 3342767.07 | 0.00 | 0.00 | 0.00 | 0.00 |
| TG 56:6\|TG 20:1_18:2_18:3 | 0.00 | 0.00 | 0.00 | 0.00 | 0.00 | 0.00 | 0.00 | 0.00 | 2194385.11 | 2038006.99 |
| TG 56:6\|TG 9:0_9:0_38:6 | 0.00 | 0.00 | 0.00 | 0.00 | 0.00 | 0.00 | 571136.70 | 503311.64 | 0.00 | 0.00 |
| TG 57:0\|TG 16:0_17:0_24:0 | 2874642.16 | 2738221.86 | 2768709.61 | 2811828.07 | 3030375.49 | 2850248.62 | 6643725.28 | 4537318.44 | 3079156.00 | 2713941.36 |
| TG 57:1\|TG 16:0_25:0_16:1 | 2402521.51 | 2254152.99 | 2439447.51 | 2485190.72 | 2713301.27 | 2681663.20 | 5654957.36 | 3858187.50 | 2864146.89 | 2557870.10 |
| TG 57:2\|TG 23:0_16:1_18:1 | 1857271.35 | 1734256.70 | 1832587.18 | 1637502.54 | 1696056.53 | 2211974.37 | 2923174.72 | 2147054.38 | 1758395.11 | 2002402.91 |
| TG 57:3\|TG 16:1_17:1_24:1 | 0.00 | 0.00 | 0.00 | 0.00 | 775934.97 | 1359065.64 | 1157656.70 | 835013.91 | 0.00 | 0.00 |
| TG 57:3\|TG 21:0_18:1_18:2 | 9209529.95 | 7946020.62 | 9280146.74 | 9059209.72 | 8747815.95 | 10062204.64 | 0.00 | 0.00 | 7873438.22 | 8226688.16 |
| TG 57:4\|TG 21:0_18:2_18:2 | 0.00 | 0.00 | 0.00 | 0.00 | 570154.05 | 856724.53 | 0.00 | 0.00 | 0.00 | 0.00 |
| TG 58:0\|TG 16:0_17:0_25:0 | 2393130.81 | 2353071.13 | 1669812.04 | 2341389.83 | 2521490.87 | 1632672.04 | 4584531.32 | 2854714.53 | 1835911.22 | 1457484.95 |
| TG 58:1\|TG 16:0_24:0_18:1 | 4180337.95 | 4331198.35 | 4072760.33 | 3732323.43 | 3940678.90 | 6704145.30 | 5796086.42 | 3822127.50 | 3850901.00 | 5245823.59 |
| TG 58:2\|TG 16:0_24:0_18:2 | 0.00 | 0.00 | 607533.98 | 462102.43 | 7459216.88 | 16678200.77 | 0.00 | 0.00 | 0.00 | 0.00 |
| TG 58:2\|TG 22:0_18:1_18:1 | 9586376.65 | 8849378.97 | 8093858.12 | 5534068.51 | 0.00 | 0.00 | 0.00 | 0.00 | 6853497.33 | 10532887.77 |
| TG 58:2\|TG 24:0_16:1_18:1 | 630391.62 | 592607.53 | 0.00 | 0.00 | 0.00 | 0.00 | 4168349.43 | 2840671.56 | 501420.83 | 690424.32 |
| TG 58:3\|TG 22:0_18:1_18:2 | 8357611.19 | 7810808.56 | 7414419.83 | 4973994.20 | 5223054.10 | 12494839.83 | 1378239.06 | 1125268.98 | 4910528.83 | 7241963.79 |
| TG 58:4\|TG 18:1_22:1_18:2 | 6901745.73 | 6050320.41 | 5604312.49 | 4101620.77 | 4556131.79 | 9722822.54 | 610267.92 | 618610.16 | 3936040.44 | 5690217.48 |
| TG 58:4\|TG 22:0_18:2_18:2 | 201506.16 | 221886.29 | 291009.17 | 249801.11 | 243375.38 | 434611.82 | 610267.92 | 618610.16 | 277414.61 | 351602.86 |
| TG 58:5\|TG 18:0_18:0_22:5 | 0.00 | 0.00 | 0.00 | 0.00 | 0.00 | 0.00 | 0.00 | 0.00 | 0.00 | 0.00 |
| TG 58:5\|TG 22:1_18:2_18:2 | 1414554.81 | 1226747.63 | 1368796.24 | 1223480.77 | 866304.10 | 1951535.47 | 0.00 | 0.00 | 787735.83 | 1306043.30 |
| TG 59:0\|TG 16:0_17:0_26:0 | 957233.84 | 976096.91 | 0.00 | 0.00 | 1043181.97 | 1525858.34 | 3392020.76 | 1391382.81 | 1100020.11 | 955515.83 |
| TG 59:0\|TG 16:0_18:0_25:0 | 0.00 | 0.00 | 954914.59 | 869342.32 | 0.00 | 0.00 | 0.00 | 0.00 | 0.00 | 0.00 |
| TG 59:1\|TG 16:0_25:0_18:1 | 1560431.24 | 1526526.80 | 1528405.30 | 1388860.55 | 1450329.13 | 1586658.79 | 3384251.32 | 2123088.91 | 1496795.89 | 1406489.32 |
| TG 59:2\|TG 25:0_16:1_18:1 | 1470290.70 | 1337637.01 | 1467575.58 | 1294104.75 | 1401359.88 | 1862537.46 | 2439991.13 | 1511515.00 | 1376007.44 | 1545027.48 |
| TG 59:3\|TG 23:0_18:1_18:2 | 783982.97 | 710229.02 | 723381.44 | 553548.67 | 549786.71 | 1183691.38 | 0.00 | 0.00 | 682276.06 | 856145.83 |
| TG 59:3\|TG 25:0_16:1_18:2 | 0.00 | 0.00 | 0.00 | 0.00 | 0.00 | 0.00 | 673948.87 | 504537.34 | 0.00 | 0.00 |
| TG 59:4\|TG 23:0_18:2_18:2 | 405079.68 | 417782.16 | 425056.13 | 307144.14 | 293283.01 | 610535.03 | 0.00 | 0.00 | 273975.61 | 395376.31 |
| TG 60:0\|TG 16:0_18:0_26:0 | 951306.92 | 522207.53 | 988109.39 | 508854.70 | 523875.72 | 538803.59 | 1444178.21 | 1122082.66 | 1056497.33 | 516022.18 |
| TG 60:0\|TG 17:0_18:0_25:0 | 0.00 | 0.00 | 0.00 | 0.00 | 0.00 | 0.00 | 987230.09 | 403768.59 | 0.00 | 0.00 |
| TG 60:1\|TG 16:0_26:0_18:1 | 1286176.65 | 1203832.99 | 1158447.74 | 1036466.85 | 1285218.84 | 1896571.49 | 2134283.02 | 1289416.33 | 1281413.78 | 1534911.85 |
| TG 60:2\|TG 24:0_18:1_18:1 | 5433491.03 | 5553806.13 | 5349400.99 | 3788932.38 | 4381732.37 | 10991061.55 | 2125278.30 | 1488096.72 | 3960253.06 | 7340978.45 |
| TG 60:3\|TG 24:0_18:1_18:2 | 7440376.87 | 6788767.27 | 6118322.43 | 3914176.80 | 4979977.05 | 12663211.49 | 1040054.43 | 802540.47 | 4645554.56 | 8238374.95 |
| TG 60:4\|TG 24:0_18:2_18:2 | 6538025.62 | 6017059.38 | 5856287.40 | 3777305.53 | 4132442.54 | 10764873.65 | 300757.36 | 388741.02 | 3826357.78 | 6412097.09 |
| TG 60:5\|TG 24:0_18:2_18:3 | 902396.54 | 864399.85 | 823715.75 | 547055.08 | 659320.58 | 1228096.69 | 0.00 | 0.00 | 565402.11 | 878357.18 |
| TG 61:0\|TG 16:0_17:0_28:0 | 0.00 | 0.00 | 0.00 | 0.00 | 0.00 | 0.00 | 853796.60 | 615954.30 | 0.00 | 0.00 |
| TG 61:0\|TG 16:0_20:0_25:0 | 329900.92 | 334737.53 | 383351.60 | 315915.64 | 357323.35 | 395238.56 | 0.00 | 0.00 | 373307.72 | 323023.06 |
| TG 61:1\|TG 16:0_27:0_18:1 | 544145.73 | 501279.48 | 483010.11 | 471513.15 | 541827.63 | 547550.00 | 1264592.36 | 758837.89 | 539815.11 | 471778.01 |
| TG 61:2\|TG 25:0_18:1_18:1 | 705743.51 | 758292.53 | 0.00 | 0.00 | 624026.47 | 857076.63 | 0.00 | 0.00 | 629291.72 | 777250.92 |
| TG 61:2\|TG 27:0_16:1_18:1 | 0.00 | 0.00 | 706413.65 | 573504.81 | 0.00 | 0.00 | 1153547.17 | 755475.31 | 0.00 | 0.00 |
| TG 61:3\|TG 25:0_18:1_18:2 | 498138.27 | 488398.51 | 452809.72 | 373640.00 | 457825.03 | 800646.24 | 0.00 | 0.00 | 437440.22 | 578237.33 |
| TG 61:4\|TG 25:0_18:2_18:2 | 319316.86 | 281498.66 | 282504.53 | 146642.10 | 214268.84 | 541638.34 | 0.00 | 0.00 | 222738.89 | 363359.81 |
| TG 62:0\|TG 16:0_22:0_24:0 | 301521.68 | 280125.93 | 217465.19 | 228121.22 | 261060.00 | 212368.90 | 563941.51 | 467372.11 | 280615.00 | 240943.20 |
| TG 62:1\|TG 16:0_28:0_18:1 | 465004.86 | 462434.02 | 409985.30 | 405316.35 | 396146.47 | 565034.14 | 1359839.62 | 584308.91 | 450030.89 | 505923.16 |
| TG 62:2\|TG 16:0_16:1_30:1 | 0.00 | 0.00 | 0.00 | 0.00 | 0.00 | 0.00 | 0.00 | 0.00 | 0.00 | 0.00 |
| TG 62:2\|TG 26:0_18:1_18:1 | 1152569.62 | 1210666.60 | 1150729.28 | 920593.87 | 1128601.85 | 2337245.97 | 0.00 | 0.00 | 1137282.17 | 1829764.47 |
| TG 62:3\|TG 26:0_18:1_18:2 | 1370298.81 | 1394980.93 | 1393673.92 | 925419.61 | 1215390.93 | 2947537.85 | 528233.40 | 394073.52 | 1124847.11 | 2209797.67 |
| TG 62:4\|TG 26:0_18:2_18:2 | 1240655.14 | 1072106.50 | 990850.39 | 584385.08 | 886854.74 | 2290878.90 | 0.00 | 0.00 | 868611.83 | 1544206.89 |
| TG 62:5\|TG 26:0_18:2_18:3 | 0.00 | 0.00 | 0.00 | 0.00 | 181930.46 | 339586.63 | 0.00 | 0.00 | 196479.50 | 215037.86 |
| TG 63:0\|TG 16:0_23:0_24:0 | 0.00 | 0.00 | 0.00 | 0.00 | 0.00 | 0.00 | 372697.36 | 275774.92 | 0.00 | 0.00 |
| TG 63:1\|TG 15:0_16:0_32:1 | 205427.73 | 238678.09 | 190758.29 | 207401.82 | 230179.31 | 252122.54 | 506807.83 | 473713.75 | 243859.67 | 193413.45 |
| TG 63:2\|TG 15:0_16:1_32:1 | 0.00 | 0.00 | 0.00 | 0.00 | 286183.47 | 336145.69 | 0.00 | 0.00 | 273669.22 | 274130.58 |
| TG 63:2\|TG 15:0_18:1_30:1 | 262921.95 | 242117.53 | 0.00 | 0.00 | 0.00 | 0.00 | 655530.75 | 459969.45 | 0.00 | 0.00 |
| TG 63:3\|TG 27:0_18:1_18:2 | 194464.86 | 180156.70 | 0.00 | 0.00 | 0.00 | 0.00 | 0.00 | 0.00 | 0.00 | 0.00 |
| TG 64:1\|TG 16:0_24:0_24:1 | 187988.38 | 257472.78 | 0.00 | 0.00 | 0.00 | 0.00 | 0.00 | 0.00 | 0.00 | 0.00 |
| TG 64:1\|TG 16:0_30:0_18:1 | 0.00 | 0.00 | 0.00 | 0.00 | 155393.24 | 200984.75 | 627623.02 | 303790.08 | 266579.89 | 238204.66 |
| TG 64:2\|TG 16:0_16:1_32:1 | 317135.95 | 306355.77 | 315526.74 | 222452.27 | 0.00 | 0.00 | 491006.98 | 410151.09 | 0.00 | 0.00 |
| TG 64:2\|TG 28:0_18:1_18:1 | 0.00 | 0.00 | 0.00 | 0.00 | 375119.08 | 470317.24 | 0.00 | 0.00 | 315254.28 | 402860.68 |
| TG 64:3\|TG 16:1_18:1_30:1 | 0.00 | 0.00 | 0.00 | 0.00 | 0.00 | 0.00 | 0.00 | 0.00 | 0.00 | 0.00 |
| TG 64:3\|TG 28:0_18:1_18:2 | 369554.27 | 424624.69 | 293436.35 | 245970.94 | 392937.23 | 527766.57 | 0.00 | 0.00 | 290206.89 | 443792.82 |
| TG 64:4\|TG 28:0_18:2_18:2 | 189088.11 | 208652.94 | 0.00 | 0.00 | 212441.62 | 360019.72 | 0.00 | 0.00 | 205848.78 | 371620.05 |
| TG 66:2\|TG 16:0_18:1_32:1 | 0.00 | 0.00 | 0.00 | 0.00 | 0.00 | 0.00 | 0.00 | 0.00 | 143536.00 | 140287.38 |
| TG 66:3\|TG 30:0_18:1_18:2 | 0.00 | 0.00 | 0.00 | 0.00 | 152126.82 | 216450.22 | 0.00 | 0.00 | 0.00 | 0.00 |
