# Supplementary material for: Lipidomic Profiling of Rice Bran after Green Solid–Liquid Extractions for the Development of Circular Economy Approaches
Source: Foods. 2023 Jan 13;12(2):384. doi: 10.3390/foods12020384 (PMC9857567; doi:10.3390/foods12020384)
Supplement: Supplementary file 1 [file foods-12-00384-s001.zip › Table S4.pdf]

Table S4

Legend	
**Abbreviation**	**Name**
**ET**	Ethanol (99%) at 4 °C
**ET20**	Ethanol (99%) at 20 °C
**WSBU**	Water-saturated 1-butanol at 4 °C
**CH-ME**	Chloroform/methanol (2:1, v/v) at 4 °C
**MTBE-ME**	Methyl tert-butyl ether/methanol (3:1, v/v) at 4 °C
**NAE**	N-acylethanolamine
**ST**	Sterol

The Relative Abundances of NAEs and STs in different methods

Name	ET SAMPLE 1	ET SAMPLE 2	ET20 SAMPLE 1	ET20 SAMPLE 2	WSBU SAMPLE 1	WSBU SAMPLE 2	CH-ME SAMPLE 1	CH-ME SAMPLE 2	MTBE-ME SAMPLE 1	MTBE-ME SAMPLE 2
**NAE 13:1**	1108858.92	356714.95	0.00	0.00	49446.47	141367.02	0.00	0.00	208064.11	139309.17
**NAE 14:0**	487744.00	592019.48	0.00	0.00	0.00	0.00	1368059.87	249962.42	751890.28	124829.90
**NAE 14:1**	270512.00	1116668.97	304893.26	138352.27	138186.88	370235.58	258864.34	777113.87	475779.72	281078.84
**NAE 16:0**	0.00	0.00	0.00	0.00	0.00	0.00	0.00	0.00	285278.67	15953.93
**NAE 20:1**	1306079.35	940290.93	308399.34	154653.98	94039.02	312815.25	196029.76	747958.75	303189.56	205009.17
**NAE 21:1**	0.00	0.00	0.00	0.00	0.00	0.00	0.00	0.00	103621.11	98272.09
**NAE 22:4**	143689.46	517697.89	0.00	0.00	87028.38	161328.07	699069.34	544157.73	96125.28	93358.35
**ST 29:1;O;Hex;FA 16:0**	403533.19	295901.49	312283.76	419116.13	334756.30	432501.88	0.00	0.00	620679.89	493095.92
**ST 29:1;O;Hex;FA 18:1**	428959.57	313672.37	276877.90	380920.55	291646.88	387789.28	0.00	0.00	576841.83	429323.59
**ST 29:1;O;Hex;FA 18:2**	268029.30	210397.11	188105.69	181503.98	180389.83	232602.43	0.00	0.00	353958.00	328551.70
