# Supplementary figures and images for: Lipidomic Profiling of Rice Bran after Green Solid–Liquid Extractions for the Development of Circular Economy Approaches
Source: Foods. 2023 Jan 13;12(2):384. doi: 10.3390/foods12020384 (PMC9857567; doi:10.3390/foods12020384)

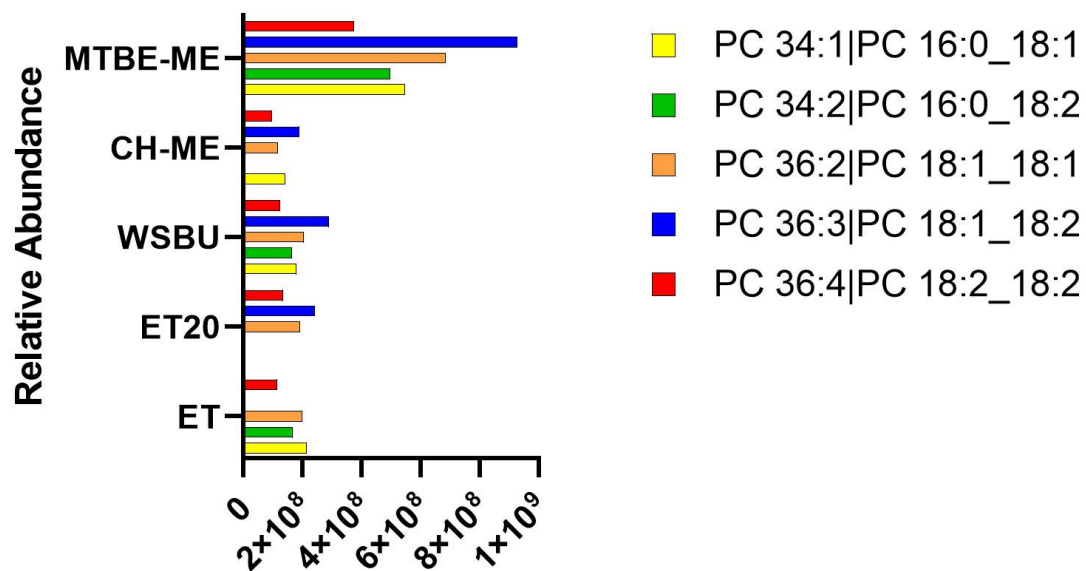

**Figure S1.** Relative abundance of main PCs acyl chains in different extraction methods.

Supplement: Supplementary file 1 [file foods-12-00384-s001.zip › Figure S1.pdf]
